# The genome sequence of Small-leaved Sweet-briar, *Rosa agrestis* Savi (Rosales: Rosaceae)

**DOI:** 10.12688/wellcomeopenres.27851.1

**Published:** 2026-09-18

**Authors:** Maarten J. M. Christenhusz, Royal Botanic Gardens Kew Genome Acquisition Lab, Plant Genome Sizing Collective, Darwin Tree of Life Barcoding collective, Wellcome Sanger Institute Tree of Life Management, Samples and Laboratory team, Wellcome Sanger Institute Scientific Operations: Sequencing Operations, Wellcome Sanger Institute Tree of Life Core Informatics team, Tree of Life Core Informatics collective, Darwin Tree of Life Consortium

**Affiliations:** 1 Royal Botanic Gardens Kew, Richmond, Surrey, England, UK; 2 Botanical Society of Britain and Ireland, St Albans, Hertfordshire, England, UK

**Keywords:** Rosa agrestis, Small-leaved Sweet-briar, genome sequence, chromosomal, Rosales

## Abstract

We present a genome assembly of *Rosa agrestis* (Small-leaved Sweet-briar; Streptophyta; Magnoliopsida; Rosales; Rosaceae). The chromosome complement (2 *n* = 5 *x* = 35) is represented across two haplotypes with total lengths of 1,939.70 megabases and 450.00 megabases. Most of the haplotype 1 assembly (99.34%) was scaffolded into 28 chromosomal pseudomolecules. Most of haplotype 2 (97.6%) is scaffolded into 7 chromosomal pseudomolecules. Together, the two assemblies represent all 35 chromosomes. The mitochondrial genome assembly has a length of 271.19 kilobases and the plastid genome assembly has a length of 156.55 kilobases. Gene annotation of the haplotype 1 assembly by Ensembl identified 83,865 protein-coding genes. This assembly was generated as part of the Darwin Tree of Life project, which produces reference genomes for eukaryotic species found in Britain and Ireland.

## Species taxonomy

Eukaryota; Viridiplantae; Streptophyta; Streptophytina; Embryophyta; Tracheophyta; Euphyllophyta; Spermatophyta; Magnoliopsida; Mesangiospermae; eudicotyledons; Gunneridae; Pentapetalae; rosids; fabids; Rosales; Rosaceae; Rosoideae; Rosoideae incertae sedis; *Rosa*; *Rosa agrestis* Savi (NCBI:txid267229)

## Background

*Rosa agrestis* Savi (Small-leaved Sweet-briar) is an erect deciduous shrub with arching stems (Figure 1). Its native range extends from Europe to the Caucasus and includes north-western Africa ( POWO, 2026). In Britain and Ireland, where it is native, it occurs mainly in lowland chalk and limestone districts, in habitats including scrub, grassland, hedges, old quarries and coastal headlands ( Maskew, 2023). Populations often comprise only a few plants. It is most frequent in central Ireland and rare in southern Britain, where increased recording has revealed few additional sites. Local losses have resulted from ploughing and increased shading following reduced grazing; scrub clearance can also threaten surviving populations ( Maskew, 2023).

*Rosa agrestis* belongs to the dog rose group ( *Rosa* section Caninae), and both pentaploid (2 *n* = 5 *x* = 35) and hexaploid (2 *n* = 6 *x* = 42) plants have been reported ( Herklotz *et al.*, 2018). The specimen sequenced here is pentaploid. Dog roses have an unusual reproductive system known as canina meiosis. In pentaploid plants, 14 chromosomes pair to form seven bivalents, while the remaining 21 remain unpaired as univalents. Pollen transmits seven chromosomes and egg cells transmit 28, including all 21 univalents. This asymmetric inheritance restores pentaploidy following fertilisation ( Herklotz *et al.*, 2025).

We sequenced the genome of *Rosa agrestis* as part of the Darwin Tree of Life Project, which aims to generate high-quality reference genomes for all named eukaryotic species in Britain and Ireland to support research, conservation, and the sustainable use of biodiversity. The sequenced specimen was collected from Kew Gardens, Surrey, UK.

**Figure 1.**
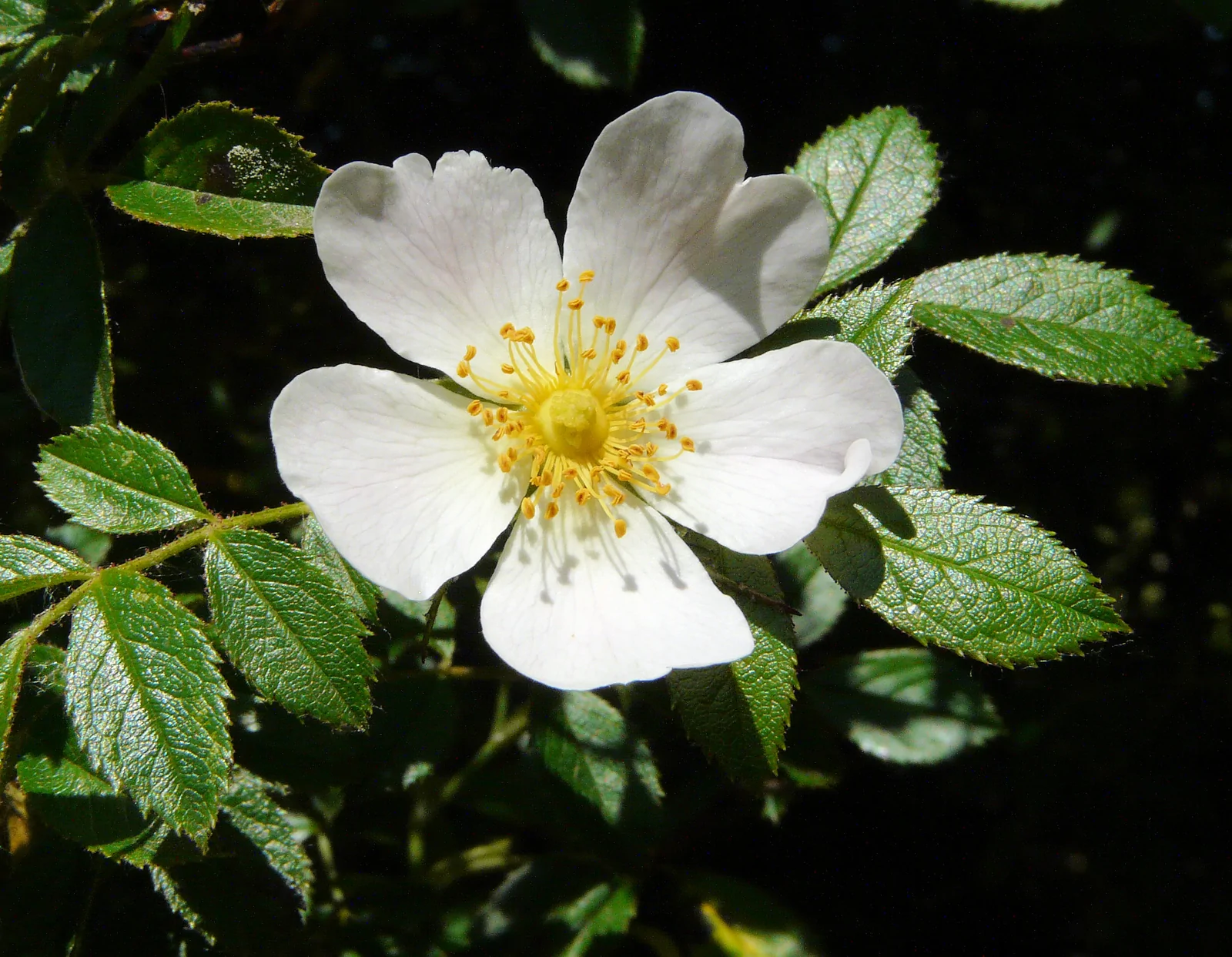
*R. agrestis* photograph by Andrea Moro.

## Methods

### Sample acquisition, flow cytometry and DNA barcoding

A specimen of *R. agrestis* (specimen ID KDTOL10315, ToLID drRosAgre1) was used for genome sequencing. It was collected from Kew Gardens, Surrey, UK (latitude 51.4774, longitude −0.2922) on 2021-07-26. The specimen was collected and identified by Maarten Christenhusz. The drRosAgre1 specimen was also used for Hi-C and RNA sequencing. Sample metadata record the herbarium voucher for the sequenced plant (collector number MC9245) as specimen K001400911 in the Herbarium, Royal Botanic Gardens, Kew (K). A duplicate herbarium voucher from the same collection is deposited in the Herbarium, Royal Botanic Garden Edinburgh (E), as specimen E01152855.

The genome size was estimated by flow cytometry following the ‘one-step’ method outlined in Pellicer *et al*. (2021) and using propidium iodide as the fluorochrome. The General Purpose Buffer (GPB) supplemented with 3% PVP was used for isolation of nuclei ( Loureiro *et al.*, 2007), and the internal calibration standard was *Petroselinum crispum* ‘Champion Moss Curled’ with an assumed 1C-value of 2,200 Mb ( Obermayer *et al.*, 2002).

The initial identification was verified by an additional DNA barcoding process according to the framework developed by Twyford *et al*. (2024). Part of the plant specimen was preserved in silica gel desiccant ( Chase & Hills, 1991). DNA extracted from the dried plant was amplified by PCR for standard barcode markers, with the amplicons sequenced and compared to public sequence databases including GenBank and the Barcode of Life Database (BOLD) ( Ratnasingham & Hebert, 2007). Following whole genome sequence generation, the relevant DNA barcode region was also used alongside the initial barcoding data for sample tracking at the WSI ( Twyford *et al.* , 2024). The standard operating procedures for Darwin Tree of Life barcoding are available on protocols.io.

### Nucleic acid extraction

High molecular weight (HMW) DNA extraction protocols developed by the Tree of Life Core Laboratory at the Wellcome Sanger Institute (WSI) are available on protocols.io ( Howard *et al.*, 2025). Tissue from the drRosAgre1 sample was weighed and triaged to select the appropriate extraction workflow. HMW DNA was prepared for three PacBio HiFi libraries: DTOL13456494, DTOL13085700 and DTOL13456494 (ERX13030757). The tissue input, HMW DNA extraction, downstream fragmentation and clean-up, and post-shearing quality-control measurements for each PacBio library are summarised in Table 1.

The concentration of the sheared and purified DNA was assessed with a NanoDrop spectrophotometer and a Qubit Fluorometer using the Qubit dsDNA High Sensitivity Assay kit. Fragment size distribution was evaluated by running the sample on the Femto Pulse system.

**Table 1.** DNA extraction and post-extraction processing for each PacBio library used for *R. agrestis*.

**PacBio library ID**	**Tissue preparation**	**HMW DNA extraction**	**Fragmentation and clean-up**	**Post-shearing QC**
DTOL13456494	53.0 mg leaf; cryoPREP	Automated Plant MagAttract v3	Fragmentation: Megaruptor®3 for LI PacBio; clean-up: Automated SPRI	Qubit 14.5 ng/μL; fragment size 13.2 kb
DTOL13085700	58.0 mg leaf; cryoPREP	Automated Plant MagAttract v2	Fragmentation: Covaris g-TUBE for ULI PacBio; clean-up: Automated SPRI	Qubit 1.8 ng/μL; fragment size 10.2 kb; GQN 5.7
DTOL13456494 (ERX13030757)	53.0 mg leaf; cryoPREP	Automated Plant MagAttract v3	Fragmentation: Megaruptor®3 for LI PacBio; clean-up: Automated SPRI	Qubit 14.5 ng/μL; fragment size 13.2 kb

RNA was extracted from leaf tissue of drRosAgre1 in the Tree of Life Laboratory at the WSI using the RNA Extraction: Automated MagMax™ *mir*Vana protocol. The RNA concentration was assessed with a NanoDrop spectrophotometer and a Qubit Fluorometer using the Qubit RNA Broad-Range Assay kit. RNA integrity was assessed using the Agilent RNA 6000 Pico Kit and Eukaryotic Total RNA assay.

### PacBio HiFi library preparation and sequencing

Library preparation and sequencing were performed by the WSI Scientific Operations core.

Library DTOL13456494 was prepared using the SMRTbell Prep Kit 3.0 (Pacific Biosciences), following the manufacturer’s instructions. The kit includes reagents for end repair/A-tailing, adapter ligation, post-ligation SMRTbell bead clean-up and nuclease treatment. Size selection and clean-up were performed using diluted AMPure PB beads (Pacific Biosciences). DNA concentration was measured using a Qubit Fluorometer v4.0 (Thermo Fisher Scientific) with the Qubit 1X dsDNA HS assay kit. Final library fragment size was assessed using an Agilent Femto Pulse Automated Pulsed Field CE Instrument (Agilent Technologies) with the gDNA 55 kb BAC analysis kit. This libary was sequenced on a Revio instrument (Pacific Biosciences). Libraries with concentrations above 2 nM were diluted to 2 nM, and 15 μL was used for complex preparation. For libraries below 2 nM, the available 10 μL was used without dilution. Primers were annealed and polymerases were bound to the SMRTbell templates according to the manufacturer’s instructions. Complexes were purified using 1.2× SMRTbell beads, diluted to a loading concentration of 200–300 pM and supplemented with a Revio sequencing internal control. Sequencing used Revio 25M SMRT cells.

Library DTOL13085700 was prepared using the ultra-low-input (ULI) PacBio HiFi workflow, with the SMRTbell Express Template Prep Kit 2.0 and gDNA Sample Amplification Kit (Pacific Biosciences). Before library preparation, DNA was fragmented to approximately 10 kb and the input was normalised to 20 ng. Single-strand overhang removal, DNA damage repair and end repair/A-tailing were performed according to the manufacturer’s instructions, followed by adapter ligation. A 0.85× pre-PCR clean-up was performed using ProNex beads (Promega). The DNA was divided equally between two PCR reactions (A and B), which were performed according to the manufacturer’s protocol. A 0.85× post-PCR clean-up was performed using ProNex beads. DNA concentration was measured using a Qubit Fluorometer v4.0 with the Qubit HS Assay Kit, and fragment size was assessed using the Agilent Femto Pulse with the gDNA 55 kb BAC analysis kit. PCR products from reactions A and B were then pooled to provide at least 500 ng DNA in 47.4 μL. The pooled sample underwent a second round of DNA damage repair and end repair/A-tailing, followed by hairpin adapter ligation. A 1× clean-up was performed using ProNex beads, followed by DNA quantification using the Qubit and fragment size analysis using the Agilent Femto Pulse. Size selection was performed using the PippinHT system (Sage Science), with the target fragment size determined by Femto Pulse analysis (typically 4–9 kb). Size-selected libraries were cleaned using 1× ProNex beads and normalised to 2 nM before sequencing. Libraries DTOL13085700 and DTOL13456494 (ERX13030757) were sequenced across two runs on a Sequel IIe instrument (Pacific Biosciences), at loading concentrations of 40–135 pM.

For both sequencing platforms, SMRT Link software (Pacific Biosciences) was used to configure and monitor runs and perform primary and secondary data analysis.

### Hi-C

***Sample preparation and crosslinking***

Hi-C data were generated from the drRosAgre1 leaf sample using the Arima-HiC v2 kit (Arima Genomics). Tissue was finely ground using the Covaris cryoPREP Dry Pulverizer (Covaris), and then subjected to nuclei isolation. Nuclei were isolated using a modified protocol based on the Qiagen QProteome Cell Compartment Kit (Qiagen), in which only the Lysis and CE2 buffers were used, with QIAshredder spin columns. After isolation, nuclei were fixed using formaldehyde to a final concentration of 2% to crosslink the DNA. The crosslinked DNA was then digested and biotinylated according to the manufacturer’s instructions. A clean-up step was performed with SPRIselect beads before library preparation. DNA concentration was quantified using the Qubit Fluorometer v4.0 (Thermo Fisher Scientific) and the Qubit HS Assay Kit, following the manufacturer’s instructions.

***Hi-C library preparation and sequencing***

Biotinylated DNA constructs were fragmented using a Covaris E220 sonicator and size-selected to 400–600 bp using SPRISelect beads. DNA was enriched with Arima-HiC v2 kit Enrichment beads. End repair, A-tailing, and adapter ligation were carried out with the NEBNext Ultra II DNA Library Prep Kit (New England Biolabs), following a modified protocol where library preparation occurs while DNA remains bound to the Enrichment beads. Libraries were amplified with KAPA HiFi HotStart mix and a custom Unique Dual Index (UDI) barcode set (Integrated DNA Technologies). Depending on sample concentration and biotinylation percentage determined at the crosslinking stage, libraries were amplified with 10–16 PCR cycles. Post-PCR clean-up was performed with SPRISelect beads. Libraries were quantified using the AccuClear Ultra High Sensitivity dsDNA Standards Assay Kit (Biotium) and a FLUOstar Omega plate reader (BMG Labtech).

Prior to sequencing, libraries were normalised to 10 ng/μL. Normalised libraries were quantified again to create equimolar and/or weighted 2.8 nM pools. Pool concentrations were checked using the Agilent 4200 TapeStation (Agilent) with High Sensitivity D500 reagents before sequencing. Libraries were sequenced on the Illumina NovaSeq 6000, generating paired-end 150-bp reads.

### RNA library preparation and sequencing

Libraries were prepared with the NEBNext® Ultra II Directional RNA Library Prep Kit for Illumina (New England Biolabs), following the manufacturer’s instructions. Poly(A) mRNA was isolated from 100 ng of total RNA using oligo(dT) beads, converted to cDNA, and uniquely indexed; 14 PCR cycles were performed. Libraries were prepared with an intended average fragment size of 100–300 bp. Libraries were quantified, normalised, and pooled equimolarly to a final concentration of 2.8 nM before dilution to 150 pM for loading. Sequencing was carried out on the Illumina NovaSeq 6000, generating paired-end reads.

### Genome assembly

Prior to assembly of the PacBio HiFi reads, a database of *k*-mer counts ( *k* = 31) was generated from the filtered reads using FastK. The *k*-mer frequency distribution was analysed with GenomeScope (version 2.1.0) ( Ranallo-Benavidez *et al.*, 2020), providing fitted point estimates of genome size, heterozygosity and repeat content.

The HiFi reads were assembled using Hifiasm in Hi-C phasing mode ( Cheng *et al.*, 2021, 2022), producing two haplotypes. Hi-C reads ( Rao *et al.* , 2014) were mapped to contigs using bwa-mem2 ( Vasimuddin *et al.*, 2019). Contigs were further scaffolded with Hi-C data in YaHS ( Zhou *et al.*, 2023), using the --break option for handling potential misassemblies. The scaffolded assemblies were evaluated using Gfastats ( Formenti *et al.* , 2022), BUSCO ( Manni *et al.*, 2021) and MerquryFK ( Rhie *et al.*, 2020). The organelle genomes were assembled using OATK ( Zhou *et al.*, 2025). The comparator for the mitochondrial assembly was the mitochondrial genome of *Rosa* hybrid cultivar (NC_077627.1).

### Assembly curation

The assembly was decontaminated using the Assembly Screen for Cobionts and Contaminants ( ASCC) pipeline. The flat files and maps for curation were generated with TreeVal. Manual curation was conducted primarily in PretextView and HiGlass ( Kerpedjiev *et al.* , 2018). Scaffolds were visually inspected and corrected as described by Howe *et al*. (2021). The two haplotype assemblies were combined for curation. Manual corrections included 34 breaks and 95 joins. This reduced the scaffold count by 12.9%, increased the scaffold N50 by 5.1%, and increased the total assembly length by 37.5%. The curation process is described at sanger-tol/curation-resources. A Hi-C contact map of the haplotype 1 assembly was generated with PretextSnapshot.

### Assembly quality assessment

The MerquryFK tool ( Rhie *et al.*, 2020) was run in a Singularity container ( Kurtzer *et al.*, 2017) to evaluate *k*-mer completeness and assembly quality for both haplotypes using the *k*-mer database ( *k* = 31) computed prior to genome assembly. The analysis outputs included assembly QV scores and completeness statistics.

The haplotype 1 assembly was assessed using BlobToolKit ( Challis *et al.*, 2020). PacBio reads were mapped to the assembly to derive sequence coverage, and BUSCO ( Manni *et al.*, 2021) was used to assess gene-set completeness. DIAMOND BLASTp searches of extracted BUSCO proteins and DIAMOND BLASTx searches of assembly sequences against UniProt Reference Proteomes ( Buchfink *et al.*, 2021), followed by BLASTn searches of sequences without DIAMOND BLASTx hits against the NCBI nt database ( Altschul *et al.*, 1990), supported taxonomic assignment. Coverage, sequence composition, taxonomic assignment and BUSCO results were integrated into a BlobDir for interactive inspection and visualisation. The snail and blob plots were rendered locally from this hosted BlobDir using BlobTk v0.8.3. The scaffold-length radial scale function for the snail plot was set to linear ( Challis & Blaxter, 2026). The blob plot used circular markers with area proportional to scaffold length ( Challis *et al.*, 2020).

## Genome sequence report

### Sequence data

The genome of a specimen of *R. agrestis* was sequenced using Pacific Biosciences single-molecule HiFi long reads, generating 95.61 Gb (gigabases) from 8.49 million reads. We assembled the genome from these reads. GenomeScope analysis using a *p* = 2 model gave a *k*-mer-based haploid genome size estimate of 1,194.02 Mb, with heterozygosity of 1.69% and repeat content of 73.52%; the full-model fit was 87.02% (Figure 2). Using flow cytometry, the genome size (1C‐value) of the sample was estimated to be 1.48 pg, equivalent to 1,450.00 Mb. These estimates guided expectations for the assembly. The NCBI assembly record reports 77× genome coverage. Hi-C sequencing produced 110.61 Gb from 366.24 million read pairs, which were used to scaffold the haplotype 1 assembly. RNA sequencing data were also generated and are available in public sequence repositories. Table 2 summarises the specimen and sequencing details.

**Figure 2.**
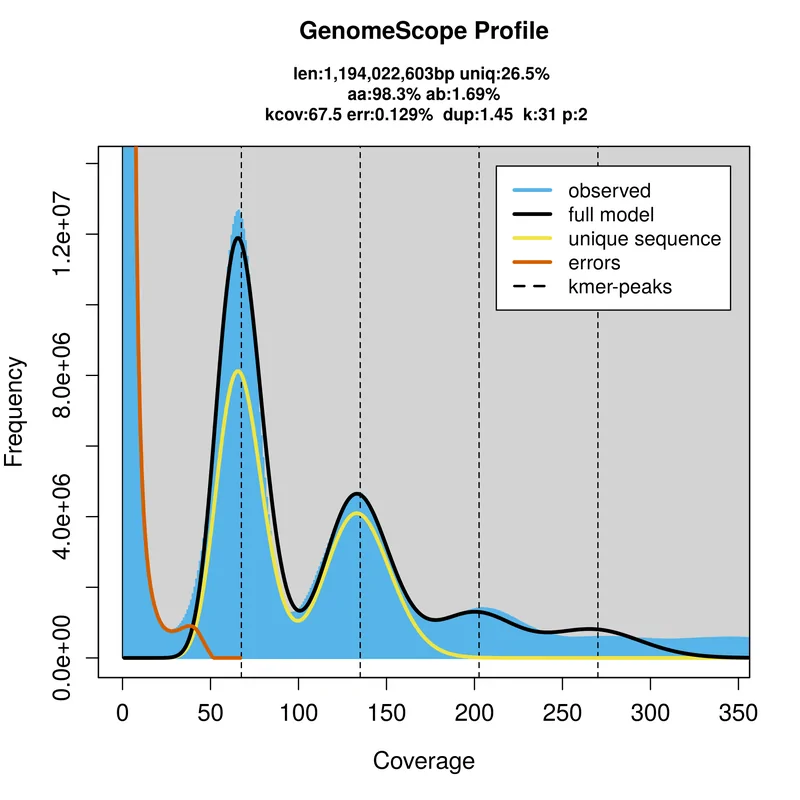
GenomeScope model fitted to the *k*-mer frequency distribution. The plot shows the observed and modelled *k*-mer spectra used to estimate genome size, heterozygosity and repeat content from unassembled sequencing reads.

**Table 2.** Specimen and sequencing data for *R. agrestis* (BioProject PRJEB79880)

**Platform**	**PacBio HiFi**	**Hi-C**	**RNA-seq**
**ToLID**	drRosAgre1	drRosAgre1	drRosAgre1
**Specimen ID**	KDTOL10315	KDTOL10315	KDTOL10315
**BioSample (source individual)**	SAMEA10369461	SAMEA10369461	SAMEA10369461
**BioSample (tissue)**	SAMEA10369505	SAMEA10369505	SAMEA10369505
**Tissue**	leaf	leaf	leaf
**Instrument**	Revio (1 run); Sequel IIe (2 runs)	Illumina NovaSeq 6000	Illumina NovaSeq 6000
**Library ID & run accession(s)**	DTOL13456494: ERR13660053; DTOL13085700: ERR13660054; DTOL13456494 (ERX13030757): ERR13660055	ERR13670011	ERR13670012
**Read count total**	8.49 million reads	366.24 million read pairs	39.83 million read pairs
**Base count total**	95.61 Gb	110.61 Gb	12.03 Gb

### Assembly statistics

The genome was assembled into two haplotypes using Hi-C phasing. The haplotype 1 assembly was curated to chromosome level, and haplotype 2 was also scaffolded to chromosome level. The haplotype 1 assembly, which is proposed as the reference assembly for this species, has a total length of 1,939.70 Mb in 254 scaffolds (excluding organelle sequences), with 595 gaps, and a scaffold N50 of 71.9 Mb (Table 3). The haplotype 1 assembly has a scaffold auN of 70.4 Mb.

**Table 3.** Genome assembly statistics for *R. agrestis*

**Genome assembly**	**Haplotype 1**	**Haplotype 2**
**Assembly name**	drRosAgre1.hap1.1	drRosAgre1.hap2.1
**Assembly accession**	GCA_964662855.1	GCA_964662835.1
**Assembly level**	chromosome	chromosome
**Span (Mb)**	1,939.70	450.00
**Number of chromosomes**	28	7
**Number of contigs**	849	275
**Contig N50 (Mb)**	5.46	7.04
**Number of scaffolds (excluding organelle sequences)**	254	162
**Scaffold N50 (Mb)**	71.9	59.68
**Scaffold auN (Mb)**	70.4	62.5
**Longest scaffold length (Mb)**	92.73	76.39
**Organelles**	Mitochondrial genome: 271.19 kb; Plastid genome: 156.55 kb	-

The sample is pentaploid (2 *n* = 5 *x* = 35) and has unusual asymmetric meiosis called ‘canina meiosis’, where 7 pairs of chromosomes recombine and the remaining 21 are inherited as univalents through the ovum. Recombining pairs of chromosomes were identified based on sequence similarity (inferred from the Hi‑C contact map). One set of 7 putatively recombining chromosomes was placed in the haplotype 1 (primary) assembly alongside the 21 univalents. The other set of 7 putatively recombining chromosomes have been placed in the haplotype 2 assembly. In the haplotype 1 assembly, the seven putatively recombining chromosomes are numbered 1–7 in decreasing order of length, and the 21 univalent chromosomes are numbered 8–28 separately in decreasing order of length (Figure 3; Table 4).

The mitochondrial genome (length 271.19 kb, OZ215701.1) and plastid genome (length 156.55 kb, OZ215702.1) were also assembled. These sequences are included as contigs in the multifasta file of the haplotype 1 genome submission and as standalone records.

**Figure 3.**
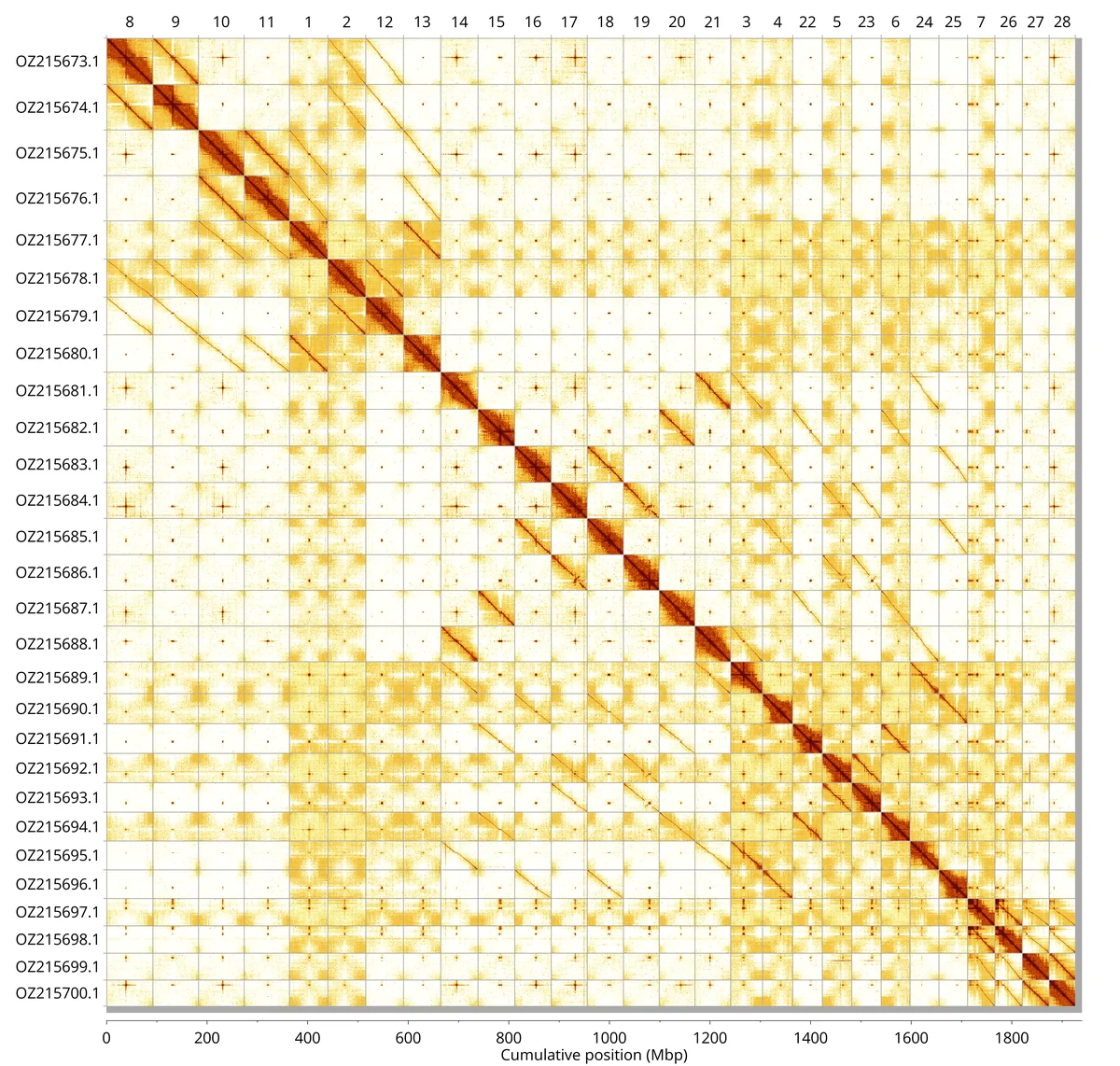
Hi-C contact map of the *R. agrestis* haplotype 1 assembly. Assembled chromosomes are shown in order of size and labelled along the axes, with a megabase scale shown below. The plot was generated using PretextSnapshot.

**Table 4.** Chromosomal pseudomolecules in the haplotype 1 genome assembly of *R. agrestis* drRosAgre1 (haplotype 2 also at chromosome level)

**INSDC accession**	**Molecule**	**Length (Mb)**	**GC%**
OZ215677.1	1	76.20	39
OZ215678.1	2	75.37	39.50
OZ215689.1	3	63.74	39
OZ215690.1	4	59.87	39.50
OZ215692.1	5	58.59	39
OZ215694.1	6	57.59	39
OZ215697.1	7	54.55	39.50
OZ215673.1	8	92.73	39
OZ215674.1	9	90.67	39
OZ215675.1	10	90.59	39
OZ215676.1	11	90.04	39
OZ215679.1	12	74.96	39
OZ215680.1	13	74.47	39
OZ215681.1	14	73.62	39
OZ215682.1	15	73.52	38.50
OZ215683.1	16	72.12	39
OZ215684.1	17	71.90	39
OZ215685.1	18	71.90	39
OZ215686.1	19	71.17	38.50
OZ215687.1	20	70.89	39
OZ215688.1	21	70.84	39
OZ215691.1	22	59.01	39
OZ215693.1	23	58.04	39
OZ215695.1	24	57.48	39.50
OZ215696.1	25	56.83	39
OZ215698.1	26	54.28	39.50
OZ215699.1	27	53.40	39
OZ215700.1	28	52.50	39

### Assembly quality metrics

For the haplotype 1 assembly, the estimated QV is 67.9, and for the haplotype 2 assembly, 65.3. When the two haplotypes are combined, the assembly achieves an estimated QV of 67.3. The *k*-mer completeness is 98.72% for the haplotype 1 assembly, 36.27% for the haplotype 2 assembly, and 98.85% for the combined haplotypes (Figure 4).

BUSCO analysis using the eudicots_odb10 reference set ( *n* = 2,326) identified 98.9% of the expected gene set (single = 1.2%, duplicated = 97.7%) in the haplotype 1 assembly. For the haplotype 2 assembly, BUSCO v.5.5.0 analysis identified 98.2% of the expected gene set (single = 93.4%, duplicated = 4.9%).

**Figure 4.**
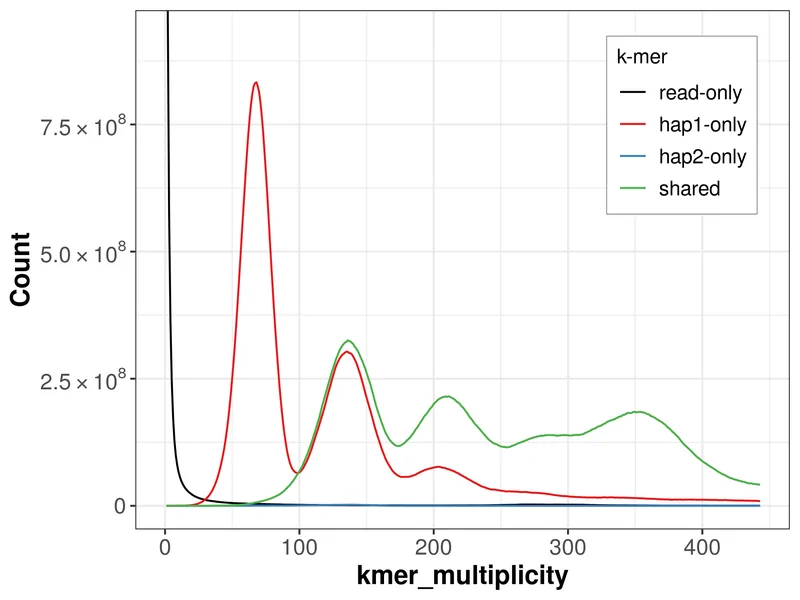
Evaluation of *k*-mer completeness using MerquryFK. This plot illustrates the recovery of *k*‐mers from the original read data in the haplotype 1 assembly, the haplotype 2 assembly and the combined haplotypes. The horizontal axis represents *k*‐mer multiplicity, and the vertical axis shows the number of *k*‐mers. The black curve represents *k*‐mers that appear in the reads but are not assembled, while the remaining curves correspond to *k*‐mers recovered in the assemblies shown.

The snail plot in Figure 5 provides a summary of assembly contiguity, cumulative scaffold count, sequence composition and BUSCO completeness for the haplotype 1 assembly ( Challis & Blaxter, 2026). The relative sizes of the longest scaffold, scaffold N50 and N90, together with the dark-grey scaffold-length distribution, show how much of the assembly is contained in long scaffolds. The outer composition track shows variation in GC, AT and N content, while the central track shows the cumulative scaffold count and the BUSCO inset summarises gene-set completeness.

The blob plot in Figure 6 displays scaffolds from the haplotype 1 assembly by GC proportion and base coverage. Marker area is proportional to scaffold length, and colour indicates taxonomic assignment ( Challis *et al.*, 2020).

**Figure 5.**
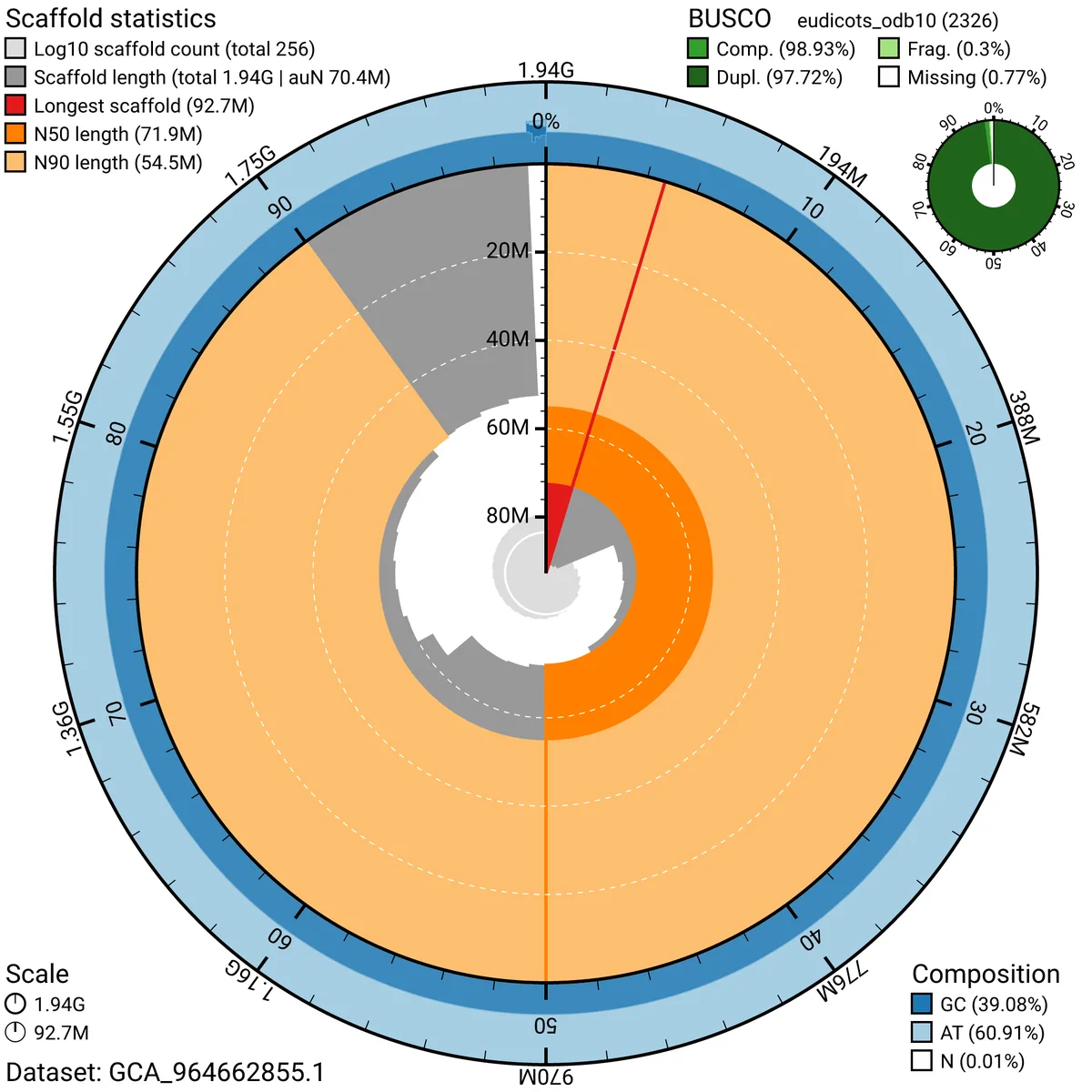
Assembly metrics for drRosAgre1.hap1.1, the haplotype 1 assembly. The BlobToolKit snail plot provides an overview of assembly metrics and BUSCO gene completeness. The circumference represents the length of the haplotype 1 assembly, and the main plot is divided into 1,000 bins around the circumference. The outermost blue tracks display the distribution of GC, AT, and N percentages across the bins. Scaffolds are arranged clockwise from longest to shortest and are depicted in dark grey. Scaffold length is shown on a linear radial scale, scaled to the longest scaffold. Red shading indicates the longest scaffold, and the deeper orange and pale orange shading represent the N50 and N90 lengths. The summary statistics include scaffold auN, the area under the scaffold Nx curve. The light grey spiral at the centre shows the cumulative scaffold count on a logarithmic scale. A summary of complete, fragmented, duplicated, and missing BUSCO genes in the eudicots_odb10 set is presented at the top right. The assembly can be explored interactively in the BlobToolKit viewer.

**Figure 6.**
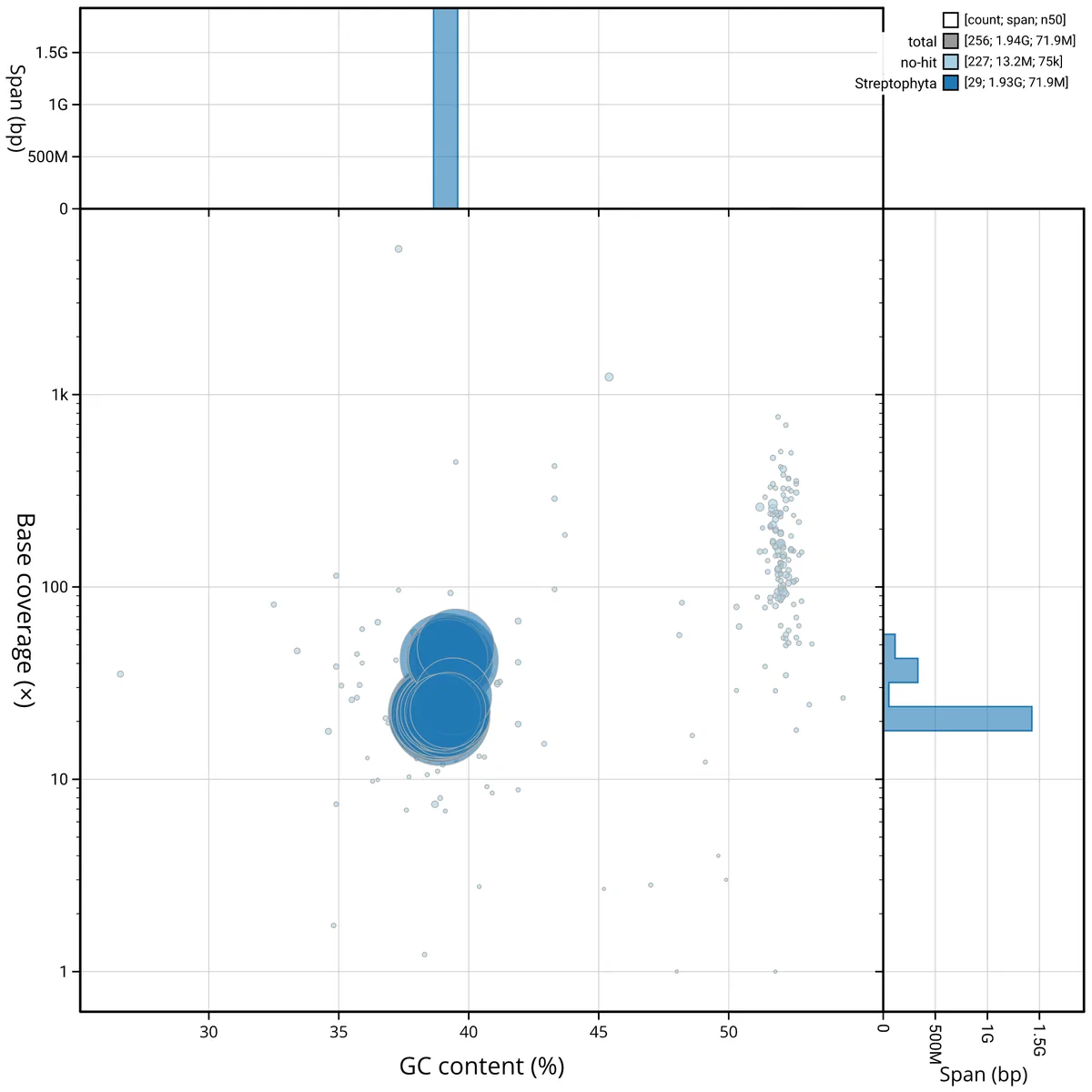
BlobToolKit blob plot for drRosAgre1.hap1.1, the haplotype 1 assembly. The plot shows base coverage (vertical axis) and GC content (horizontal axis). Circles represent scaffolds; marker area is proportional to scaffold length, and colour indicates taxonomic assignment. The histograms along the axes display the total length of sequences distributed across different levels of coverage and GC content. The assembly can be explored interactively in the BlobToolKit viewer.

Table 5 lists the assembly metric benchmarks adapted from Rhie *et al.*. (2021) and the Earth BioGenome Project (EBP) Report on Assembly Standards. The three-part EBP metric calculated for the haplotype 1 assembly is **6.C.Q67**.

**Table 5.** Earth BioGenome Project summary metrics for the *R. agrestis* assembly

**Measure**	**Value**	***Benchmark***
EBP summary (haplotype 1)	6.C.Q67	*6.C.Q40*
Contig N50 length	5.46 Mb	*> 1 Mb*
Scaffold N50 length	71.90 Mb	*= chromosome N50*
Consensus quality (QV)	Haplotype 1: 67.9; haplotype 2: 65.3; combined: 67.3	*≥ 40*
*k*-mer completeness	Haplotype 1: 98.72%; Haplotype 2: 36.27%; combined: 98.85%	*> 90%*
BUSCO	Haplotype 1: C:98.9% [S:1.2%, D:97.7%], F:0.3%, M:0.8%, n:2,326; Haplotype 2: C:98.2% [S:93.4%, D:4.9%], F:0.4%, M:1.3%, n:2,326	*S > 90%; D < 5%*
Percentage of assembly assigned to chromosomes	99.34%	*≥ 90%*

**Notes:** The EBP summary uses log10(Contig N50); chromosome-level (C) or log10(Scaffold N50); Q (Merqury QV). BUSCO v5.5.0 using the eudicots_odb10 lineage dataset: C=complete; S=single-copy; D=duplicated; F=fragmented; M=missing; n=orthologues.

### Genome annotation report

The *R. agrestis* genome assembly (GCA_964662855.1) was annotated by Ensembl at the European Bioinformatics Institute (EBI). The annotation comprises 130,697 transcripts from 83,865 protein-coding genes and 24,611 non-coding genes. The average transcript length is 2,583.97 bp, with an average of 1.20 coding transcripts per gene and 4.74 exons per transcript. For further information about the annotation, please refer to the annotation page on Ensembl.

### Data availability

European Nucleotide Archive: Rosa agrestis. Accession number PRJEB79880; https://identifiers.org/ena.embl/PRJEB79880. The genome sequence is released openly for reuse. The *R. agrestis* genome sequencing initiative is part of the Darwin Tree of Life Project ( PRJEB40665) and the Sanger Institute Tree of Life Programme ( PRJEB43745). All raw sequence data and the haplotype assemblies have been deposited in INSDC databases. Raw data and assembly accession identifiers are reported in Table 2 and Table 3.

Pipelines used for genome assembly at the WSI Tree of Life are available at https://pipelines.tol.sanger.ac.uk/pipelines. Table 6 lists software versions used in this study.

**Table 6.** Software versions and sources

**Software**	**Version**	**Source**
BLAST	2.14.0	https://ftp.ncbi.nlm.nih.gov/blast/executables/blast+/
BlobToolKit	4.3.9	https://github.com/genomehubs/blobtoolkit
BlobTk (hosted BlobDir)	0.5.1	https://github.com/genomehubs/blobtk
BlobTk (snail and blob plot renderer)	0.8.3	https://github.com/genomehubs/blobtk
BUSCO	5.5.0; 6.1.0	https://gitlab.com/ezlab/busco
bwa-mem2	2.2.1	https://github.com/bwa-mem2/bwa-mem2
DIAMOND	2.1.8	https://github.com/bbuchfink/diamond
fasta_windows	0.2.4	https://github.com/tolkit/fasta_windows
FastK	1.2	https://github.com/thegenemyers/FASTK
GenomeScope	2.1.0	https://github.com/tbenavi1/genomescope2.0
Gfastats	1.3.6	https://github.com/vgl-hub/gfastats
Hifiasm	0.19.8-r603	https://github.com/chhylp123/hifiasm
HiGlass	1.13.4	https://github.com/higlass/higlass
MerquryFK	1.1.0-c1	https://github.com/thegenemyers/MERQURY.FK
Minimap2	2.24-r1122	https://github.com/lh3/minimap2
Oatk	0.9	https://github.com/c-zhou/oatk
MultiQC	1.14; 1.17 and 1.18	https://github.com/MultiQC/MultiQC
Nextflow	23.10.0	https://github.com/nextflow-io/nextflow
PretextSnapshot	0.0.6	https://github.com/sanger-tol/PretextSnapshot
PretextView	1.0.3	https://github.com/sanger-tol/PretextView
samtools	1.19.2	https://github.com/samtools/samtools
sanger-tol/ascc	0.1.0	https://github.com/sanger-tol/ascc
sanger-tol/blobtoolkit	0.6.0	https://github.com/sanger-tol/blobtoolkit
sanger-tol/curationpretext	1.4.2	https://github.com/sanger-tol/curationpretext
Seqtk	1.4-r122	https://github.com/lh3/seqtk
Singularity	3.9.0	https://github.com/sylabs/singularity
TreeVal	1.1.1	https://github.com/sanger-tol/treeval
YaHS	1.2a.2	https://github.com/c-zhou/yahs

### Author information

- Members of the Royal Botanic Gardens Kew Genome Acquisition Lab
- Members of the Plant Genome Sizing Collective
- Members of the Darwin Tree of Life Barcoding collective
- Members of the Wellcome Sanger Institute Tree of Life Management, Samples and Laboratory team
- Members of Wellcome Sanger Institute Scientific Operations – Sequencing Operations
- Members of the Wellcome Sanger Institute Tree of Life Core Informatics team
- Members of the Tree of Life Core Informatics collective
- Members of the Darwin Tree of Life Consortium

### Wellcome Sanger Institute – Legal and Governance

The materials that have contributed to this genome note have been supplied by a Darwin Tree of Life Partner. The submission of materials by a Darwin Tree of Life Partner is subject to the **‘Darwin Tree of Life Project Sampling Code of Practice’**, which can be found in full on the Darwin Tree of Life website. By agreeing with and signing up to the Sampling Code of Practice, the Darwin Tree of Life Partner agrees they will meet the legal and ethical requirements and standards set out within this document in respect of all samples acquired for, and supplied to, the Darwin Tree of Life Project. Further, the Wellcome Sanger Institute employs a process whereby due diligence is carried out proportionate to the nature of the materials themselves, and the circumstances under which they have been/are to be collected and provided for use. The purpose of this is to address and mitigate any potential legal and/or ethical implications of receipt and use of the materials as part of the research project, and to ensure that in doing so we align with best practice wherever possible. The overarching areas of consideration are:

- Ethical review of provenance and sourcing of the material
- Legality of collection, transfer and use (national and international)

Each transfer of samples is further undertaken according to a Research Collaboration Agreement or Material Transfer Agreement entered into by the Darwin Tree of Life Partner, Genome Research Limited (operating as the Wellcome Sanger Institute), and in some circumstances, other Darwin Tree of Life collaborators.
