# Supplementary material for: The genome sequence of Small-leaved Sweet-briar, Rosa agrestis Savi (Rosales: Rosaceae)
Source: Wellcome Open Res. 2026 Sep 18;11:703. [Version 1] doi: 10.12688/wellcomeopenres.27851.1 (PMC13695711; doi:10.12688/wellcomeopenres.27851.1)
Supplement: Peer review history

# Reviewer report: Reviewer response for version 1

**DOI:** 10.21956/wellcomeopenres.30648.r174711

**Published:** 2026-10-24

**Authors:** Qilin Yang

**Affiliations:** 1 Xinjiang Institute of Ecology and Geography (Ringgold ID: 85431), Urumqi, Xinjiang, China; 2 University of Chinese Academy of Sciences, Beijing, China

This Genome Note presents a valuable chromosome-scale assembly for the pentaploid rose *Rosa agrestis* (2n = 5x = 35). The biological significance of the species and its unusual canina meiosis are described. The study uses appropriate PacBio HiFi, Hi-C and RNA-seq methods, and the resulting assembly has high continuity, consensus accuracy and completeness. The raw data, two nuclear assemblies, organellar genomes and Ensembl annotation are available through appropriate public repositories.

 I have several points that should be clarified:

 1) The terms “haplotype 1” and “haplotype 2” may be misleading because the assemblies contain 28 and seven chromosomes, respectively, rather than two equivalent genome-wide haplotypes. The authors should define this representation more explicitly and provide the evidence used to pair chromosomes 1–7 with their counterparts in the secondary assembly.

 2) The relationship between the GenomeScope estimate (1,194 Mb), the flow-cytometry estimate (1C = 1,450 Mb) and the combined assembly span (2,389.7 Mb) requires explanation. The authors should also justify using a p = 2 GenomeScope model for a pentaploid genome.

 3) The manuscript states that manual curation increased the total assembly length by 37.5%. Since breaks and joins alone should not substantially change sequence span, pre- and post-curation statistics and the source of the additional sequence should be reported.

 4) The 97.7% duplicated BUSCO result for the primary assembly is probably expected from its multi-copy, 28-chromosome representation. This should be stated explicitly so that it is not interpreted as assembly redundancy. Similarly, the reported 83,865 protein-coding genes should not be presented as directly comparable with gene counts from haploidised assemblies.

 5) The duplicated DTOL13456494 library identifier in Table 1 should be checked and clarified.

 Overall, this is a technically strong and useful genomic resource. I recommend approval with reservations, subject primarily to clarification of the assembly representation, genome-size comparisons and curation-related increase in assembly length.

Are the datasets clearly presented in a usable and accessible format, and the assembly and annotation available in an appropriate subject-specific repository?

Yes

Are sufficient details of the sequencing and extraction, software used, and materials provided to allow replication by others?

Yes

Are the rationale for sequencing the genome and the species significance clearly described?

Yes

Are the protocols appropriate and is the work technically sound?

Yes

Reviewer Expertise:

Comparative genomics and functional genomics; molecular biology of plant stress responses

I confirm that I have read this submission and believe that I have an appropriate level of expertise to confirm that it is of an acceptable scientific standard.

# Reviewer report: Reviewer response for version 1

**Published:** 2026-09-18

**Authors:** Automated Benchmarking

**Affiliations:** 1 Wellcome Sanger Institute, Hinxton, UK

As this article is a Genome Note, Automated Benchmarking has been used to determine whether the reported assembly passes four defined checks established by the Earth BioGenome Project: the raw and assembled data are publicly available, the Earth BioGenome Project three-part metric is met, the k-mer completeness exceeds 90%, and the BUSCO score passes the stated threshold. For Rosa agrestis (drRosAgre1), all four checks passed. Data available: pass. ENA study PRJEB79880, raw-read accessions ERR13660053, ERR13660054, ERR13660055, ERR13670011, and ERR13670012, and assembly accessions GCA_964662855.1 and GCA_964662835.1 are public. Earth BioGenome Project three-part metric: pass. Haplotype 1 is reported as 6.C.Q67, meeting the 6.C.Q40 reference standard. k-mer completeness: pass. The combined haplotype assemblies have 98.85% k-mer completeness, exceeding the Earth BioGenome Project target of 90%. BUSCO: pass. Haplotype 1, which is proposed as the reference assembly for this species, has 98.9% complete BUSCOs (1.2% single-copy and 97.7% duplicated). BUSCO software version: 5.5.0; lineage dataset: eudicots_odb10. It meets the plant-specific target of greater than 90% complete BUSCOs. This decision uses values reported in the Genome Note and public accession records.

Reviewer Expertise:

NA

I confirm that I have read this submission and believe that I have an appropriate level of expertise to confirm that it is of an acceptable scientific standard.
